# EATING THE PAIN AWAY: THE USE OF FOOD AS A COPING MECHANISM IN OLDER ADULTS EXPERIENCING CHRONIC PAIN

**DOI:** 10.1093/geroni/igz038.964

**Published:** 2019-11-08

**Authors:** Alexandra Felpeto, Alexandra M Ramos, Diana Hincapie, Madison B Lenox, Rebecca Reinhardt

**Affiliations:** Nova Southeastern University, Fort Lauderdale, Florida, United States

## Abstract

Individuals experiencing pain rely on impulse to make decisions, including choices regarding food consumption (Darbor, Lench, & Carter-Sowell, 2016). This study examined whether older adults experiencing chronic pain report higher instances of emotional eating in comparison to a population of older adults not experiencing chronic pain. Data stemmed from the Midlife in the United States study was analyzed to investigate whether individuals used food as a coping mechanism for chronic pain symptoms (Ryff et al., 2017). The sample consisted of Americans aged 60 to 74 years of age. Pain conditions included: has chronic pain (n=686) and does not have chronic pain (n=1036). Results of the Independent Samples T-Test indicated that participants were found to be engaging in emotional eating when experiencing chronic pain symptoms, as hypothesized. Participants in the has chronic pain condition reported relying on food as a coping mechanism more (M= 3.66, SD= 1.87) than participants in the does not have chronic pain condition (M= 3.42, SD= 1.71); t(1370)= 2.71, p= .007, d= 0.13. Results suggest that older adults experiencing chronic pain report utilizing food as a coping mechanism more than older adults that do not experience chronic pain. These findings have health implications given the rising obesity rates associated with persistent pain. Future directions may include studies on the negative health outcomes that result from high instances of emotional eating in older adults experiencing chronic pain. Additionally, investigating alternative coping mechanisms for chronic pain would be beneficial to diminish the harmful health effects of emotional eating.

